# Network module function enrichment analysis of lung squamous cell carcinoma and lung adenocarcinoma

**DOI:** 10.1097/MD.0000000000031798

**Published:** 2022-11-25

**Authors:** Piaopiao Li, Hui Yuan, Xuemei Kuang, Tingting Zhang, Lei Ma

**Affiliations:** a College of Life Science, Shihezi University, Shihezi, China; b The First Affiliated Hospital, College of Medicine, Shihezi University, Shihezi, China.

**Keywords:** co-expression module, lung adenocarcinoma, lung squamous cell carcinoma, potential biomarker, weighted gene co-expression network analysis

## Abstract

Lung squamous cell carcinoma (LUSC) and lung adenocarcinoma (LUAD) are the two major subtypes of non-small cell lung cancer that pose a serious threat to human health. However, both subtypes currently lack effective indicators for early diagnosis.

**Methods:**

To identify tumor-specific indicators and predict cancer-related signaling pathways, LUSC and LUAD gene weighted co-expression networks were constructed. Combined with clinical data, core genes in LUSC and LUAD modules were then screened using protein-protein interaction networks and their functions and pathways were analyzed. Finally, the effect of core genes on survival of LUSC and LUAD patients was evaluated.

**Results:**

We identified 12 network modules in LUSC and LUAD, respectively. LUSC modules “purple” and “green” and LUAD modules “brown” and “pink” are significantly associated with overall survival and clinical traits of tumor node metastasis, respectively. Eleven genes from LUSC and eight genes from LUAD were identified as candidate core genes, respectively. Survival analysis showed that high expression of *SLIT3*, *ABI3BP*, *MYOCD*, *PGM5*, *TNXB*, and *DNAH9* are associated with decreased survival in LUSC patients. Furthermore, high expression of *BUB1*, *BUB1B*, *TTK,* and *UBE2C* are associated with lower patient survival.

**Conclusions:**

We found biomarker genes and biological pathways for LUSC and LUAD. These network hub genes are associated with clinical characteristics and patient outcomes and they may play important roles in LUSC and LUAD.

## 1. Introduction

Lung squamous cell carcinoma (LUSC) and lung adenocarcinoma (LUAD) are the two most important subtypes of non-small cell lung cancer^[1]^ and seriously threaten human health.^[2]^ In general, LUADs grow slower and have lower mass than LUSCs. However, LUADs tend to start metastasizing early^[3,4]^ and are insensitive to radiation and chemotherapy, while LUSCs metastasize late and are usually diagnosed at a later stage.^[5,6]^ Patients with LUSCs and LUADs have poor prognosis and 5-year survival of less than 10%.^[7]^ In addition, both subtypes lack effective early diagnosis methods.^[8]^

Gene co-expression network analysis is based primarily on a systems biology methods, clustering genes with co-expression characteristic and expressing co-regulatory characteristics of genes in the form of network modules.^[9]^ Modules usually have specific functions, but genes in each module often have similar or cooperative functions. Within the same module, there may be significant upstream and downstream regulatory relationships across different functions or signaling pathways. Network module can provide new ideas for selecting genes as tumor targets. In addition, weighted co-expression network analysis (WGCNA) is an algorithm that defines gene modules with similar expression patterns in complex diseases.^[10]^ WGCNA classifies gene expression into modules where genes in the same co-expression module are similar in expression and function.^[11]^ Moreover, functional pathways can be assessed, and potential core genes can be identified by integrating modularized key components and clinical characterization data.^[12]^ Core genes are key genes in a regulatory pathways and often affect the other genes’ expression. In recent years, WGCNA methods has been used to uncover potential biomarkers or therapeutic targets associated with various diseases and cancers, such as bronchial dysplasia,^[13]^ gastric adenocarcinoma,^[14]^ and colorectal cancer.^[15]^ Therefore, the identification of key genes associated with LUSC and LUAD using WGCNA techniques allows for a better understanding of their associated signature genes.

In the present study, we established LUSC and LUAD gene co-expression modules using weighted network analysis to identify genes that may be relevant to clinical characteristics and patient outcomes. First, we constructed LUSC and LUAD network modules using gene expression data. Secondly, combining with clinical traits, we obtained core modules revealing the functions and pathways of co-expression of core genes. Finally, we validated the core hub genes by survival analysis. Our results may provide novel insights for the treatment of LUSC and LUAD.

## 2. Materials and methods

### 2.1. Ethical statement

Ethical permission was not required in this study because all data was obtained from public databases.

### 2.2. Data preparation

We downloaded gene expression and clinical trait data of 484 LUSC and 510 LUAD cohorts from cBioPortal for Cancer Genomics (http://www.cbioportal.org/). Samples were clustered to remove outliers. We log-transformed each gene expression, calculated its absolute median, and selected the top 5000 genes expressed. Statistical analysis in this study was based on R language packages.

### 2.3. Co-expression analysis

Co-expression networks of LUSC and LUAD genes were constructed using the R package WGCNA. The steps were as follows: choose the appropriate β value, evaluate the average connectivity of each gene,^[16]^ build a Pearson-based correlation neighborhood matrix, construct a gene clustering tree diagram using a hierarchical clustering method,^[16]^ dynamically cut trees to detect highly correlated genes and define them as modules, and set the minimum number of genes in a module to 30 and label the modules with different colors.

### 2.4. Identification of clinically significant modules and core genes

Eigengene and gene significance methods were used to identify modules associated with clinical traits overall survival (OS) and tumor node metastasis (TNM). The expression matrix of gene in a module was dimensionally reduced into a vector of module eigengene.^[10]^ Module membership (MM) is the correlation between genes and module vectors, reflecting the centrality of genes in modules.^[17]^ Gene significance (GS) is the relationship between genes and clinical characteristics.^[18]^ We calculated the module eigengene matrix between modules and genes and the GS matrix between traits and genes, combining the two correlation matrices and specifying modules for further analysis.

Core genes are genes that play an important role in networks, and to some extent represent the genetic characteristics of modules.^[11]^ Core genes were screened with GS and MM. We calculated the GS (clinical traits OS and TNM) and MM of genes, and genes with |GS| > 0.2 and |MM| > 0.8 were considered as core genes.

### 2.5. Construction and analysis of PPI network

Protein interaction networks (PPIs) networks were used to mine protein-protein interactions.^[19]^ We set proteins as nodes, and the relationships between proteins as edges, and used cytoscape to build a co-expression network. We used the MCODE to identify important modules in PPIs. We used the maximum cliencentrality method in the CytoHubba plugi to further identify the top core genes.^[20]^

### 2.6. GO and KEGG enrichment analysis

We annotated biological functions using STRING 11version.^[21]^ Gene ontology (GO) terms include biological processes, molecular functions, and cellular structures. The *P* < .05 is the enrichment threshold.

### 2.7. Survival analysis and validation of core genes

The impact of gene expression on patient survival was analyzed using online software Kaplan–Meier Plotter.^[22]^ Cancer samples were divided into two groups based on gene expression levels. Genes with significant differences in survival curves between the two groups were found to be strongly associated with survival in cancer patients^[11]^ (*P* < .05).

Based on the GEO database (https://www.ncbi.nlm.nih.gov/gds/), we obtained the LUSC gene expression dataset (GSE21933) including 21 LUSC cancer samples and 21 normal samples, as well as the LUAD gene expression dataset (GSE140797) including 7 cancer samples and 7 normal samples. We evaluated differences in hub genes expression between cancer and normal samples.

## 3. Results

### 3.1. Module building for LUSC and LUAD

**Figure 1.**
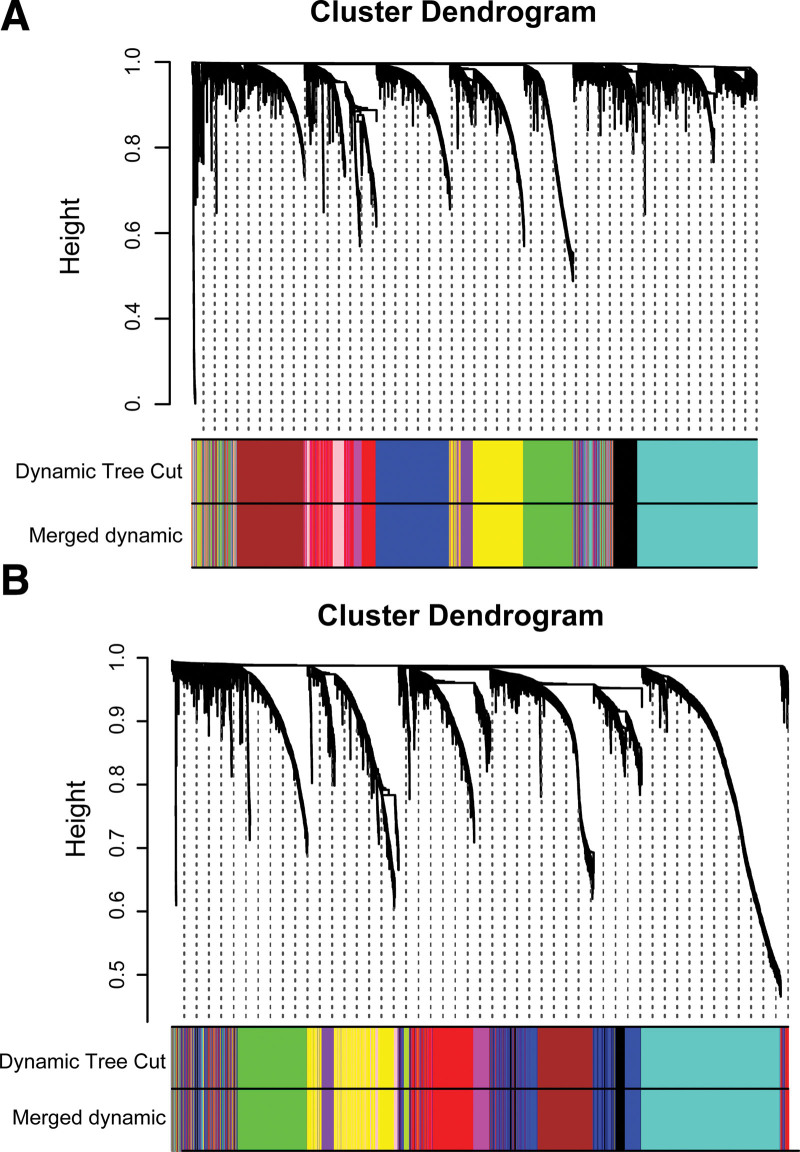
Clustering dendrogram of genes. (A, B) shows clusters of LUSC and LUAD genes, respectively. The color rows below the tree diagram show the module assignment determined by the dynamic tree cut and merged by similarity. Genes were grouped into the same module by dynamic tree cut and modules with less than 30 genes were then dynamically merged. LUAD = lung adenocarcinoma, LUSC = Lung squamous cell carcinoma.

To construct a gene co-expression network, we selected the top 5000 most expressed genes from 136 LUSC samples and 184 LUAD samples, respectively, using absolute median deviation and sample clustering methods (Fig. 1). Genes with correlation coefficient greater than 0.7 were clustered into modules (Fig. 1A, B). For visualization, we named modules with colors.

### 3.2. Modules related with clinical traits

**Figure 2.**
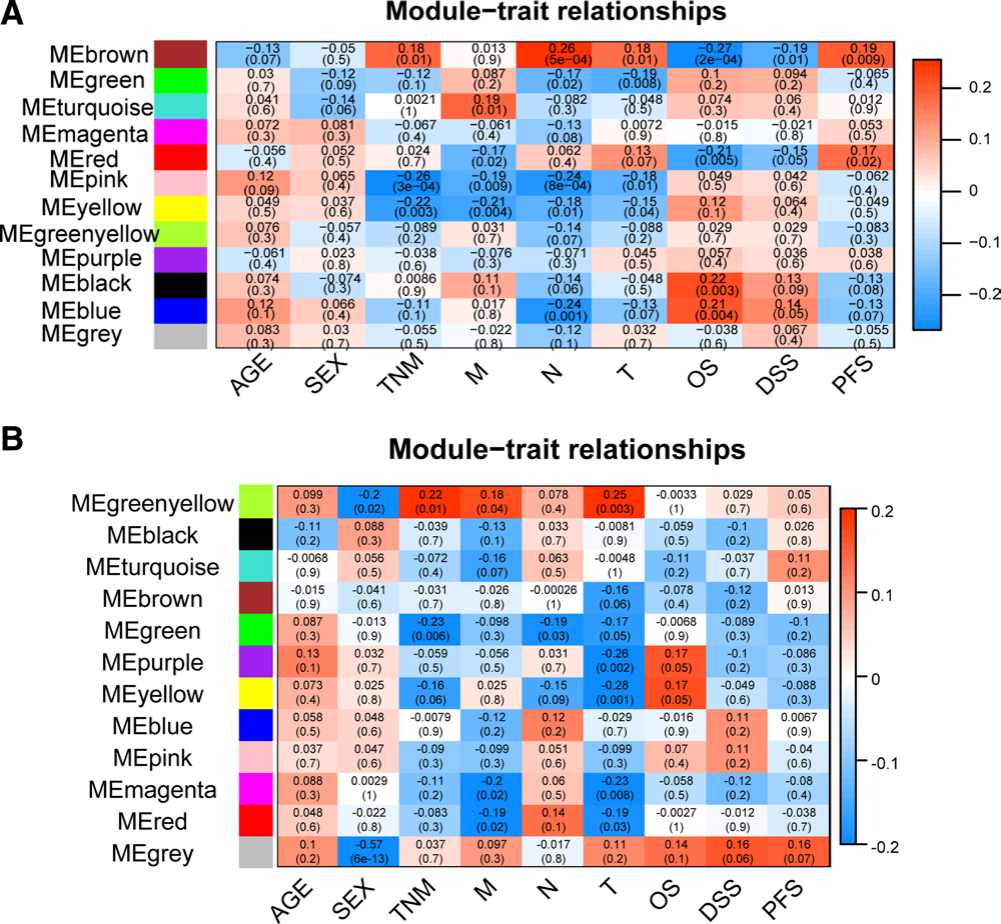
Relevance of modules to characteristics. (A, B) Correlation between modules and characteristics for LUSC and LUAD, respectively. Each row corresponds to a module eigengene, and each column corresponds to a trait. The correlation coefficient and *P* value are showed in cell. The *P* value is in parentheses. The table is color-scaled according to correlation coefficient. LUAD = lung adenocarcinoma, LUSC = Lung squamous cell carcinoma.

**Figure 3.**
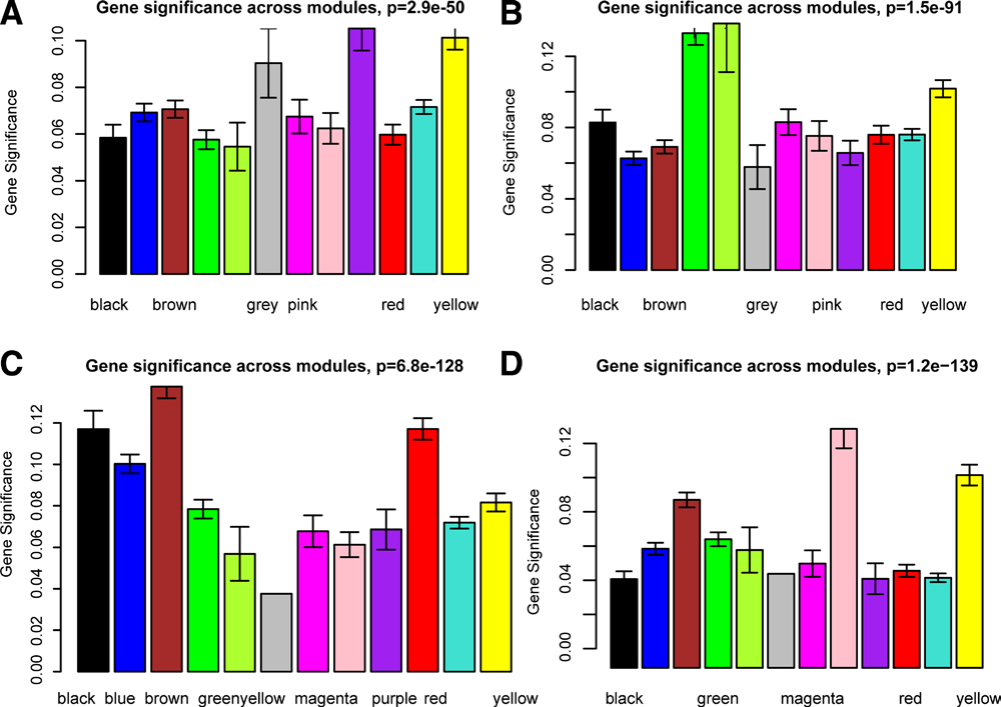
Association of genes with character-related modules. (A, C) show the associations of LUSC and LUAD genes with highly important OS-related modules, respectively. (B, D) show the associations of LUSC and LUAD genes with highly significant TNM-related modules, respectively. The color-coded bars indicate a correlation between gene expression and clinical trait data. LUAD = lung adenocarcinoma, LUSC = Lung squamous cell carcinoma, TNM = tumor node metastasis.

To further explore key modules, we correlated modules with OS and TNM. LUSC module “purple” (eigengene value = 0.17) is significantly associated with OS (Fig. 2A), and the amount of gene expression in this module is most associated with OS (Fig. 3A). LUSC module “green” (eigengene value = −0.23) module is negatively correlated with TNM, and the gene expression in this module has the highest correlation with TNM (Fig. 3B). In addition, LUAD module “brown” (eigengene value = −0.27) is significantly related with OS (Fig. 2B), and the amount of gene expression in this module has the highest correlation with OS (Fig. 3C). LUAD module “pink” (eigengene value = −0.26) is significantly related with TNM (Fig. 2B), and the amount of gene expression in this module has the highest correlation with TNM (Fig. 3D). Furthermore, LUSC modules “purple” and “green,” and LUAD modules “brown” and “pink” are most closely related to OS and TNM, implying they are clinically relevant modules for further analysis.

### 3.3. Identification of core genes

Based on MM > 0.8 and GS > 0.2, 133 genes were identified as candidate key genes, including 7 genes in the LUSC module “purple,” 58 genes in the LUSC module “green,” 68 genes in the LUAD module “brown,” and 40 genes in the LUAD module “pink.” Candidate key genes are mostly associated with PPI networks.

**Figure 4.**
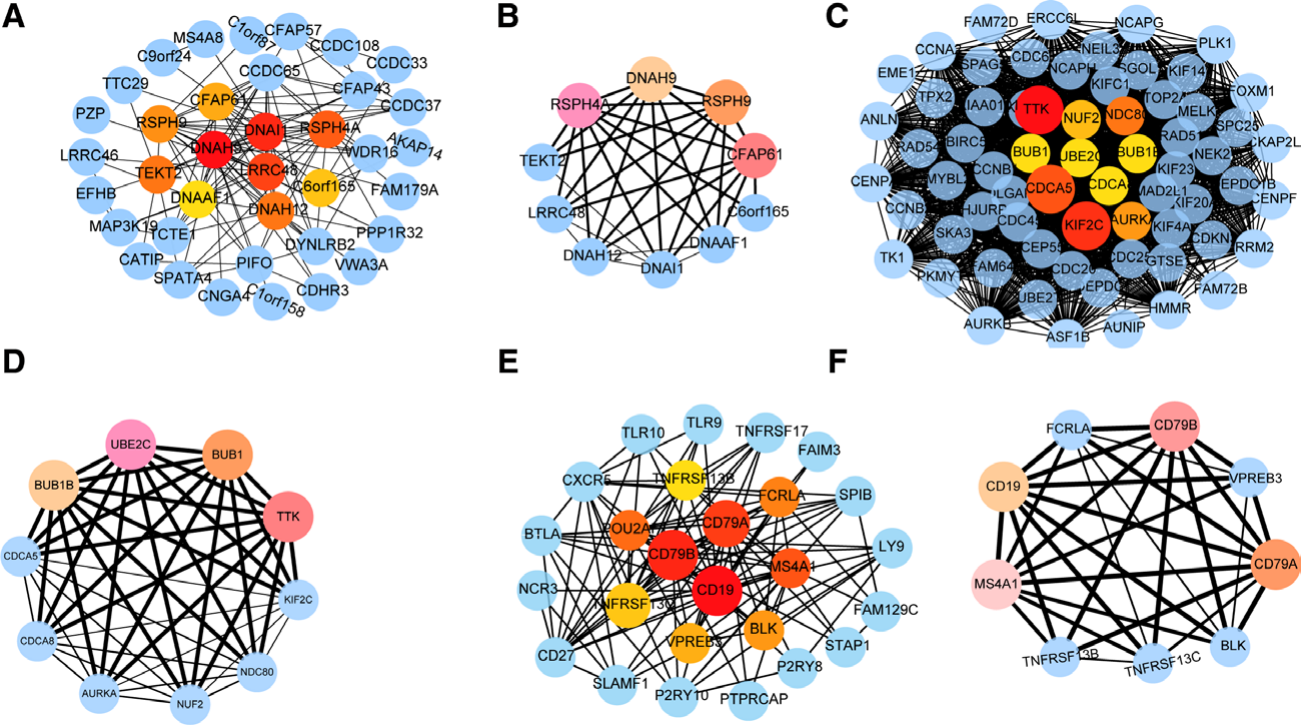
Construction and identification of PPI network. (A, C, E) shows PPI network in the LUSC module “green,” LUAD module “brown” and “pink,” respectively. (B, D, F) shows the important sub-modules of LUSC module “green,” LUAD modules “brown” and “pink” in the PPI network, respectively. Blue circles represent points in the network, while colored circles represent subnetworks that are sorted by cores. LUAD = lung adenocarcinoma, LUSC = Lung squamous cell carcinoma, PPI = protein-protein interaction network.

To further identify core genes, we constructed gene networks to determine the interactions between key genes (Fig. 4). The candidate key gene network in LUSC module “green” consists of 37 nodes (Fig. 4A). Significant parts were identified in the PPI network, consisting of 10 nodes and 32 edges. In addition, we identified four of the most important genes: *RSPH4A*, *DNAH9*, *RSPH9*, and *CFAP61* (Fig. 4B). However, candidate gene *TNXB*, *MYOCD*, *SLIT3*, *KCNA5*, *ABI3BP*, *PGM5* and *HSPB6* in the LUSC module “purple” do not constitute interaction network and may be independently involved in the LUSC development.

The candidate key gene network in LUAD module “brown” consists of 66 nodes, with a highly correlated portion of 10 nodes and 45 edges (Fig. 4C). *UBE2C*, *TTK*, *BUB1,* and *BUB1B* are the top four key genes (Fig. 4D). In addition, the candidate key gene network in LUAD module “pink” contains 26 nodes (Fig. 4E). Its most important module consists of 9 nodes and 31 edges (Fig. 4F). *CD19*, *CD79A*, *CD79B,* and *MS4A1* are the top four key genes (Fig. 4D). These genes may be potentially core genes.

### 3.4. Functional enrichment analysis for key genes

**Figure 5.**
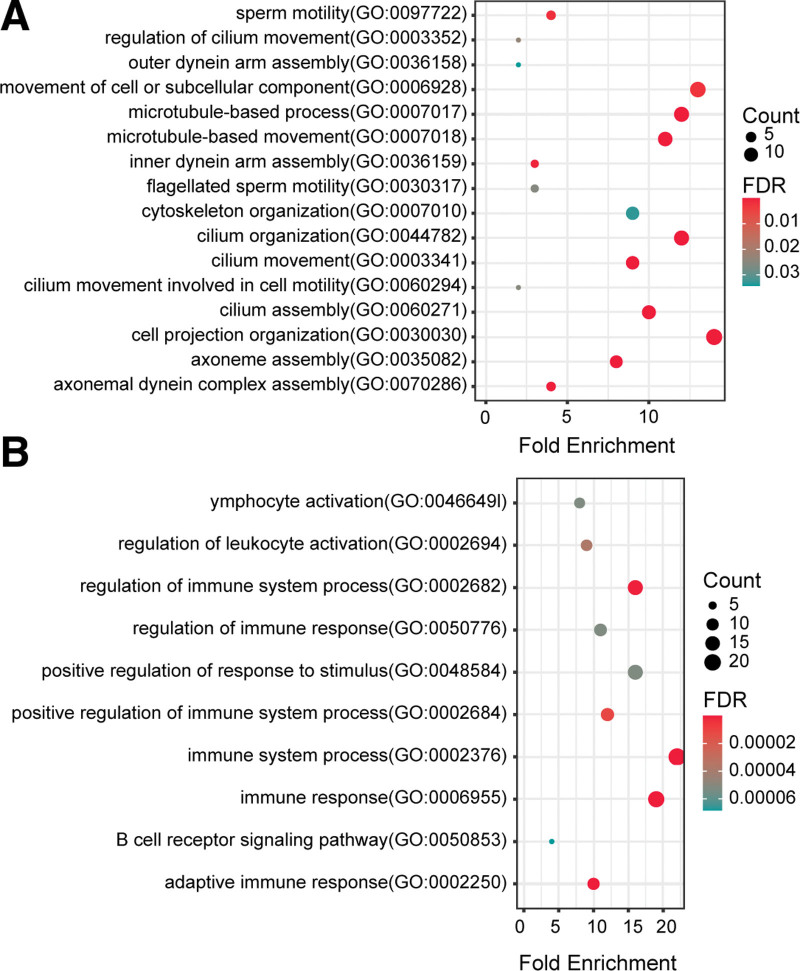
GO enrichment analysis of core genes of LUSC (A) and LUAD (B). GO = gene ontology, LUAD = lung adenocarcinoma, LUSC = Lung squamous cell carcinoma.

We performed GO functional enrichment and Kyoto Encyclopedia of Genes and Genomes (KEGG) pathway analysis on potential core genes. Different network modules are clustered in different functions and paths. For LUSC, key genes are involved in cilia motility and cell motility processes (Fig. 5A). *DNAH9* is involved in the Huntington disease pathway (see Table, Supplemental Digital Content 1, http://links.lww.com/MD/H936, which shows pathway enrichment of 11 key genes in LUSC modules “purple” and “green”). For LUAD, key genes are significantly enriched in immune response and immune system regulation (Fig. 5B). KEGG pathways analysis shows that *UBE2C*, *TTK*, *BUB1* and *BUB1B* are mainly involved in cell cycle and progesterone-mediated oocyte maturation (see Table, Supplemental Digital Content 2, http://links.lww.com/MD/H937, which shows pathway enrichment of four key genes in LUAD module “brown”). In addition, *CD19*, *CD79A*, *CD79B,* and *MS4A1* are mainly involved in the regulation of B cell receptor signaling pathway and primary immunodeficiency (see Table, Supplemental Digital Content 2, http://links.lww.com/MD/H937, which shows pathway enrichment of four key genes in LUAD module “pink”).

### 3.5. Survival analysis and validation of core genes

**Figure 6.**
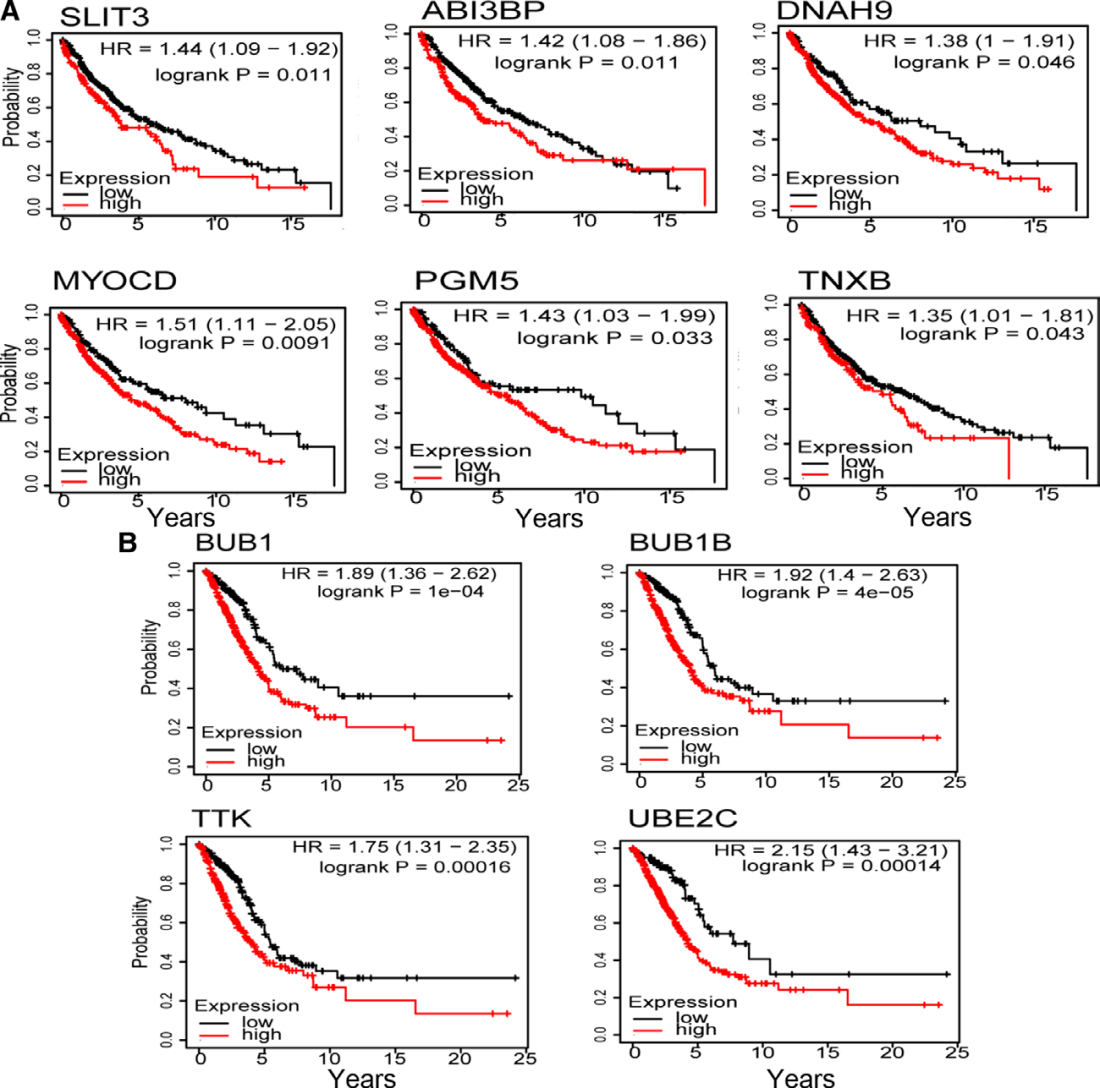
Survival curve of core genes. (A, B) Survival curve of core genes of LUSC and LUAD, respectively. Core genes expression levels have a significant impact on patient survival probability. LUAD = lung adenocarcinoma, LUSC = Lung squamous cell carcinoma.

We performed survival analysis to investigate the relationship between core genes and patients’ clinical prognosis and we found that the expression of six and four genes are strongly associated with survival in LUSC and LUAD patients, respectively (*P* < .05). High expression of *SLIT3*, *ABI3BP*, *MYOCD*, *PGM5*, *TNXB,* and *DNAH9* are associated with lower survival rate in LUSC patients, suggesting a poor prognosis (Fig. 6A). High expression of *BUB1*, *BUB1B*, *TTK,* and *UBE2C* are associated with poor survival in LUAD patients (Fig. 6B). Higher expression of these genes are associated with the worse survival in LUAD patients.

**Figure 7.**
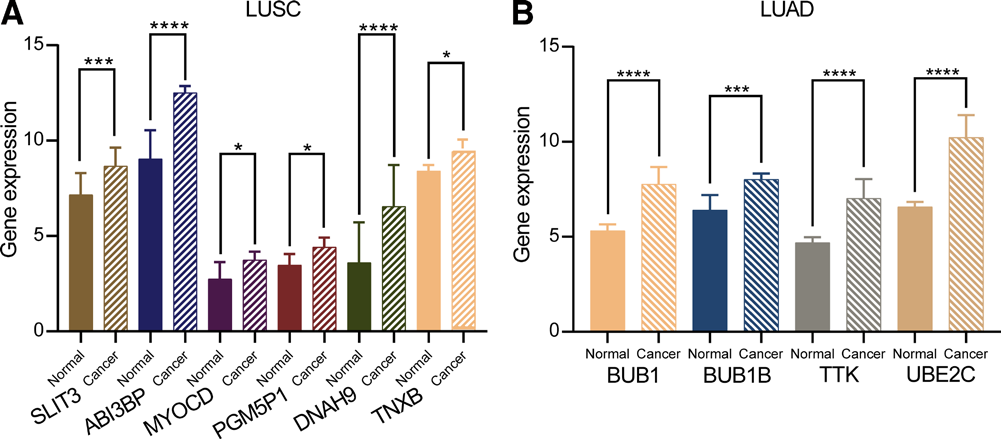
Relative expression of core genes. (A) The relative expression of *SLIT3*, *ABI3BP*, *MYOCD*, *PGM5*, *TNXB* and *DNAH*9. (B) The relative expression of *BUB1*, *BUB1B*, *TTK* and *UBE2C.* Stars represent statistical significance of *t* test: *<0.05, ***<0.001, ****<0.0001.

The expression of *SLIT3*, *ABI3BP*, *MYOCD*, *PGM5*, *TNXB*, and *DNAH9* are significantly higher in LUSC samples than in normal samples (*P* < .05, Fig. 7A). Furthermore, the expression of *BUB1*, *BUB1B*, *TTK,* and *UBE2C* genes are significantly higher in LUAD samples than in normal samples (*P* < .001, Fig. 7B). Therefore, these genes may serve as potential therapeutic targets for LUSC and LUAD, respectively.

## 4. Discussion

In the present study, we constructed weighted co-expression networks of LUSC and LUAD based on gene expression and clinical data. We identified gene modules for LUSC and LUAD. The number of genes vary widely across modules. Genes are more connected in the LUAD network than the LUSC.

We found that high expressions of *SLIT3*, *ABI3BP*, *MYOCD*, *PGM5*, *TNXB* and *DNAH9* result in reduced survival in LUSC patients. *SLIT3*, *ABI3BP*, *MYOCD*, *PGM5*, *TNXB* and *DNAH9* are strongly associated with cancer initiation and progression. These genes have important roles. For example, *SLIT3* and its regulator MiR-218-2 are simultaneously downregulated in thyroid cancer and synergistically inhibited thyroid cancer cell invasion.^[23]^ In addition, as a 3-binding protein of the ABI family, *ABI3BP* is down-regulated in esophageal cancer and inhibits the proliferation, activity, migration, and invasion of esophageal cancer cells. *ABI3BP* can serve as a potential biomarker for the diagnosis of esophageal cancer and an effective target for anti-tumor therapy.^[24]^ Furthermore, in non-small cell lung cancer, *MYOCD* overexpression drives TGF-β-induced epithelial-mesenchymal transition (EMT), which stimulates the non-small cell lung cancer cells metastasis in vivo.^[25]^ *PGM5* is a potential colorectal cancer protein marker in a large number of patients.^[26]^ Therefore, these genes may be potential drivers for LUSC cancer.

We found that high expressions of *BUB1*, *BUB1B*, *TTK* and *UBE2C* result in poor patient survival. These genes play important roles in cancer. *BUB1B* and *TTK* expression in LUAD is higher than in non-tumor lung tissue and negatively correlated with OS.^[27]^ *BUB1B* overexpression significantly reduces OS in LUAD patients.^[28]^ In addition, *UBE2C* expression is associated with poor LUAD patients’ prognosis undergoing surgery.^[29]^ In gastric cancer, overexpression of the mitotic checkpoint gene *BUB1* is associated with tumor cell proliferation and gastric cancer progression.^[30]^ These results suggest that *BUB1*, *BUB1B*, *TTK* and *UBE2C* may be targets genes for the treatment of LUAD.

## 5. Conclusions

In the present study, we constructed LUSC and LUAD gene co-expression modules using weighted network analysis and identified genes that may be relevant to clinical characteristics and patient outcomes. The 11 genes from LUSC and 8 genes from LUAD were identified as candidate core genes, respectively. Core hub gene were validated by survival analysis. High expressions of *SLIT3*, *ABI3BP*, *MYOCD*, *PGM5*, *TNXB* and *DNAH9* are related with reduced survival in LUSC patients. Furthermore, high expression of *BUB1*, *BUB1B*, *TTK* and *UBE2C* are related with lower patient survival. These core genes may play important roles in LUSC and LUAD and can be considered potential biomarkers.

## Acknowledgments

We thank all contributors of data used in the present study and the anonymous reviewers for helpful suggestions on the manuscript.

## Author contributions

PL and LM contributed to the concept and design. LM, TZ, and XK provided administrative support. PL and XK contributed to data collection and compilation. PL and HY contributed to data analysis and interpretation. PL was involved in writing the manuscript. PL and TZ provided final approval for the manuscript. All authors contributed to the article and approved the submitted version.

**Conceptualization:** Piaopiao Li, Lei Ma.

**Data curation:** Hui Yuan.

**Formal analysis:** Piaopiao Li, Hui Yuan.

**Funding acquisition:** Lei Ma.

**Investigation:** Piaopiao Li.

**Methodology:** Tingting Zhang.

**Project administration:** Tingting Zhang.

**Software:** Xuemei Kuang.

**Supervision:** Xuemei Kuang.

**Writing – original draft:** Piaopiao Li.

**Writing – review & editing:** Lei Ma.

## Supplementary Material
