# Supplementary material for: Network module function enrichment analysis of lung squamous cell carcinoma and lung adenocarcinoma
Source: Medicine (Baltimore). 2022 Nov 25;101(47):e31798. doi: 10.1097/MD.0000000000031798 (PMC9704934; doi:10.1097/MD.0000000000031798)
Supplement: Supplementary file 1 [file medi-101-e31798-s001.pdf]

Supplemental Content 1 KEGG enrichment of 11 key genes in LUSC module

Term ID	Description	FDR	Matching genes in your network
hsa05016	Huntington's disease	0.00011	DNAH9,DNAH12,DNAI1

Note: FDR, false discovery rate
