# Supplementary material for: Network module function enrichment analysis of lung squamous cell carcinoma and lung adenocarcinoma
Source: Medicine (Baltimore). 2022 Nov 25;101(47):e31798. doi: 10.1097/MD.0000000000031798 (PMC9704934; doi:10.1097/MD.0000000000031798)
Supplement: Supplementary file 2 [file medi-101-e31798-s002.pdf]

Supplemental Content 2 KEGG enrichment of 8 key genes in LUAD module

Term ID	Description	FDR	Matching genes in your network
hsa04110	Cell cycle	0.00018	BUB1B,BUB1,TTK
hsa04114	Oocyte meiosis	0.0031	AURKA,BUB1
hsa04914	Progesterone-mediated oocyte maturation	0.0031	AURKA,BUB1
hsa05340	Primary immunodeficiency	0.000000031	CD79A,TNFRSF13B,TNFRSF13C,CD19
hsa04662	B cell receptor signaling pathway	0.0000275	CD79A,CD79B,CD19
hsa04672	Intestinal immune network for IgA production	0.00072	TNFRSF13B,TNFRSF13C
hsa04640	Hematopoietic cell lineage	0.0023	MS4A1,CD19
hsa04060	Cytokine-cytokine receptor interaction	0.0138	TNFRSF13B,TNFRSF13C

Note: FDR, false discovery rate
